# Risk Factor Violations Associated With Sporadic *Salmonella* Cases

**DOI:** 10.3389/fpubh.2018.00355

**Published:** 2018-12-03

**Authors:** Xarviera S. Appling, Petrona Lee, Craig W. Hedberg

**Affiliations:** ^1^Environmental Health Division, City of Bloomington, Bloomington, MN, United States; ^2^Division of Environmental Health Sciences, School of Public Health, University of Minnesota, Minneapolis, MN, United States

**Keywords:** *Salmonella*, routine inspections, risk factor violations, FDA risk factor study, sporadic cases

## Abstract

FDA promotes assessment of routine restaurant inspection programs to measure trends in the occurrence of risk factors and compare data with national benchmarks. Reductions in the occurrence of risk factors should be accompanied by reductions in the occurrence of foodborne illnesses. The objectives of this study were to: (1) assess changes in risk factor violations in Bloomington restaurants between 2010 and 2015, in order to (2) compare patterns of risk factor violations in Bloomington restaurants that served sporadic *Salmonella* cases from 2010 to 2015 to these observed trends. FDA-based risk factor surveys were conducted in 2010 and 2015. Food contact surfaces were most frequently cited as out-of-compliance in both years. The proportion of inspections with employee health policy violations were reduced from 2010 to 2015, reflecting inspection priorities that were established after the 2010 survey. From 2010 to 2015, 154 sporadic *Salmonella* case exposures were reported from Bloomington restaurants. Food contact surfaces (odds ratio [OR] = 2.3; 95% confidence interval [CI] = 1.6, 3.5) and handwash facilities stocked (OR = 2.7; 95% CI = 1.7, 4.0) were more frequently found out-of-compliance in inspections that served sporadic *Salmonella* cases, in both individual year comparisons and in the overall comparison. The finding that violations such as food contact surfaces not being clean to sight and touch or sanitized before use and handwash facilities not being stocked with hand cleanser, sanitary towels, or hand drying devices, were more likely to be cited in sporadic *Salmonella* cases than during the FDA-based risk factor survey, suggests that these may contribute to an increased risk of *Salmonella* transmission in restaurants. Because restaurant inspections represent routine public health activities that are conducted by a broad range of agencies, establishing methods to analyze the results of these inspections across agencies and over time may help us better understand how risk factors cited on routine inspections can predict the likelihood a food establishment will be implicated in a foodborne illness case, in both sporadic cases and outbreaks.

## Introduction

The US Food and Drug Administration (FDA) is responsible for setting standards and monitoring the safe production of foods. In addition to promoting adoption of the FDA Model Food Code at the state, local and tribal level, in 1998, the FDA initiated a three-phase, 10-years study to measure the occurrence of food practices and behaviors commonly identified by the Center for Disease and Prevention (CDC) as contributing factors in foodborne illness outbreaks (1). These include improper holding temperatures, inadequate cooking, such as undercooking raw shell eggs, contaminated equipment, food from unsafe sources and poor personal hygiene (2). Results from the FDA's National Retail Food Team 10-years study have shown improvements with compliance with important safety requirements in food service and retail establishments (3). However, the study also found key practices and procedures that need further improvement in foodservice (3). These key practices include better education of food industry workers in basic food safety and restaurant procedures to prevent cross-contamination and other food handling errors that can lead to outbreaks.

To improve the performance of local food regulatory programs, the FDA works with jurisdictions to encourage enrollment in the Voluntary National Retail Food Regulatory Program Standards (RPS), a comprehensive set of nine standards that provide a foundation for program self-assessment and continuous improvement (4). A key element of the standards is a provision that the agency conducts a risk factor study on the occurrence of the five foodborne illness risk factors, at least once every 5 years to measure trends in the occurrence of the risk factors. This program assessment allows individual agencies to measure changes in performance from an initial baseline assessment, and to compare their data with the *FDA National Foodborne Illness Risk Factor Studies* (1).

Although FDA Risk Factor Studies have shown improvements in performance with respect to outbreak-associated risk factors, outbreaks are relatively uncommon events. This limits the use of outbreak prevention as a meaningful measure of retail food safety performance. We recently demonstrated that restaurants which served a sporadic case of *Salmonella* were more likely to have had inspections with observations out of compliance for prevention of contamination by hands, than were other restaurants (5). Because only 5–10% of *Salmonella* cases are associated with outbreaks, this suggests that exposures for sporadic *Salmonella* cases may be a useful measure of the effectiveness of retail food safety practices in preventing foodborne illness transmission (6).

The City of Bloomington enrolled in the Voluntary Program Standards on June 25, 2003, and conducted FDA-based risk factor surveys in 2005, 2010, and 2015. In this study, we sought to: (1) assess changes in risk factor violations in Bloomington restaurants between 2010 and 2015, in order to (2) compare patterns of risk factor violations in Bloomington restaurants that served sporadic *Salmonella* cases from 2010 to 2015 to these observed trends.

## Materials and Methods

The FDA Model Food Code prioritizes controls for risk factors and further establishes five key public health interventions to protect the health of those who eat in retail and food service establishments. These key interventions are: demonstration of knowledge, employee health controls, controlling hands as a vehicle of contamination, time and temperature parameters for controlling pathogens, and the consumer advisory (2). Based on the FDA risk factor study, 23 different risk factor observations were identified. These risk factors were: (1) Approved Source, (2) Receiving and Sound Condition, (3) Records, (4) Proper Cooking Temperature, (5) Rapid Reheating, (6) Proper Cooling, (7) Cold Holding, (8) Hot Holding at 140 degrees, (9) Time and Date Marking, (10) Separation, Segregation and Protection, (11) Food-Contact Surfaces, (12) Proper, Adequate Handwashing, (13) Good Hygienic Practices, (14) Prevention of Contamination From Hands, (15) Handwash Facilities, Chemicals, (17) Proper Cooking Temperatures, (18) Hot Holding at 135 degrees, (19) Employee Health Policy, (20) Treating Juice, (21) Cooling, Raw Shell Eggs, (22) Cold Holding, Raw Shell Eggs, and (23) Food and Food Preparation for Highly Susceptible Populations.

### City of Bloomington FDA-BASED Risk Factor Surveys

The FDA-based risk factor survey is a standardized approach used by field inspectors to document observations during routine inspections. In Bloomington and Richfield, a baseline survey was conducted in 2005, with follow-up surveys completed in 2010 and 2015 to assess the frequency of foodborne illness risk factors in food establishments in the Cities of Bloomington and Richfield, Minnesota. The surveys identified risk factors based on the 1998 FDA Food Code. Surveys in 2010 and 2015 were used to determine if there were changes in the occurrence of observed risk factors due to interventions implemented by the City following the previous survey.

Each inspector was asked to fill out the FDA baseline data collection form (survey) for the establishments selected in their territory. The survey was completed based on observations at the time of the inspection. The survey consisted of 51 total observations. The survey was broken into 23 different risk factor observations and characterized into six different risk factor groups. The six groups included food from an unsafe source, inadequate cooking, improper holding, contaminated equipment, poor personal hygiene, and other, which included chemical hazards. The inspectors marked violations as being IN, OUT, NO (not observed), and NA (not applicable). Data collected from the surveys was then entered into two separate Microsoft Access databases, i.e., one for the City of Bloomington data and one for the Richfield data. The databases were created to match the data collection form provided by FDA.

For this study, four reports were run to extract the 2010 and 2015 FDA-based survey data. The 2010 Bloomington and Richfield data were merged into one excel document. The 2015 Bloomington and Richfield data were also merged into one document. The City's electronic inspection software program defaults all violations to IN compliance unless otherwise noted by inspection. As a result, items marked NO and NA for the FDA risk factor study were all marked IN compliance for the purpose of this study. The proportions of inspections with observations out of compliance in 2010 were compared to the proportions of inspections with observations out of compliance in 2015.

### Sporadic *Salmonella* Case Reports

In Minnesota, *Salmonella* infections are required by law to be reported to the Minnesota Department of Health (MDH). Reported cases are interviewed by MDH staff about potential exposures during the 7 days before illness onset, including all food establishments patronized. The MDH maintains surveillance data for all laboratory-confirmed *Salmonella* cases. Restaurant exposure histories for de-identified sporadic *Salmonella* cases between 2010 and 2015 in the Cities of Bloomington and Richfield, were obtained from MDH. One hundred fifty-four food establishments were identified that served *Salmonella* cases that were not associated with outbreaks.

Risk factors violations from restaurants that served a sporadic *Salmonella* case were exported from the health inspection reports corresponding to the inspection nearest to the exposure date of the case. Risk factors were broken into the same categories as in the FDA-based risk factor study; 23 different risk factor observations and characterized into six different risk factor groups. For evaluation of sporadic case exposures, the proportions of case inspections with observations out of compliance were compared to the proportions of the FDA-based survey with observations out of compliance (7). Three sets of comparisons were made. Inspections from sporadic cases reported in 2010 and 2015 were compared to the FDA–based survey results from the corresponding years, and inspection results for all cases reported from 2010 to 2015 were compared to the combined FDA-based survey results from 2010 to 2015 (7).

## Results

### FDA-Based Survey Comparison

The 2010 survey included 156 restaurants (80 fast food and 76 full service), while the 2015 survey included 155 restaurants (84 fast food and 71 full service). In both of these years, the percentage of full-service restaurants in the survey exceeded the percentage in the licensed inventory by 3–4%. Of the 23 total FDA risk factor violations assessed during the routine inspections, the percentage marked out of compliance ranged from zero to 37.4%. Eight were rarely cited out of compliance.

Of the 15 risk factors that were cited, three were observed out-of-compliance more frequently in 2015 than in 2010; one was observed less frequently (Table 1). In both years, food contact surfaces were most frequently cited as out-of-compliance, based on observations that food contact surfaces and utensils were not clean to sight and touch or sanitized prior to use. In 2010, food contact surfaces were out-of-compliance in 23.7% of inspections compared to 37.4% in 2015. Cold holding violations were observed in 12.8% of inspections in 2010 and in 29% of inspections in 2015. Handwash facilities being accessible during routine inspections was out-of-compliance in 2.6% of inspections in 2010 and 15.5% of inspections in 2015. Employee health policy was observed out-of-compliance in 20.5% of inspections in 2010, but only in 4.5% of inspections in 2015.

**Table 1:** Comparison of risk factor observations in FDA-based surveys conducted by the City of Bloomington, 2010 and 2015.

**FDA risk factor**	**2010 FDA-based survey (156 Food establishments)**	**2015 FDA-based survey (155 Food establishments)**	**Comparison between 2015 and 2010 surveys**
	**No. (%) Inspections with violation out of compliance**	**No. (%) Inspections with violation out of compliance**	**Odds ratio (95% Confidence interval)**
Certified food manager	28 (17.9)	28 (18.1)	1.0 (0.6, 1.8)
Receiving conditions	1 (0.6)	0 (0)	Undefined
Cold holding	20 (12.8)	45 (29)	2.8 (1.6, 5.0)
Hot holding	10 (6.4)	15 (9.7)	1.6 (0.7, 3.6)
Time as a public health control	6 (3.9)	14 (9.0)	2.4 (0.9, 6.6)
Separate raw from ready to eat foods	18 (11.5)	25 (16.1)	1.5 (0.8, 2.8)
Food protected environmental contamination	5 (3.2)	5 (3.2)	1.0 (0.3, 3.5)
Food contact surfaces	37 (23.7)	58 (37.4)	1.9 (1.2, 3.1)
Proper, adequate handwashing	10 (6.4)	8 (5.2)	0.8 (0.3, 2.1)
Good hygienic practices	11 (7.1)	5 (3.2)	0.4 (0.1, 1.3)
Prevent contamination by hands	16 (10.3)	10 (6.5)	0.6 (0.3, 1.4)
Handwash facilities accessible	4 (2.6)	24 (15.5)	7.0 (2.4, 20.6)
Handwash facilities stocked	29 (18.6)	38 (24.5)	1.4 (0.8, 2.8)
Chemicals properly identified/stored/used	14 (9.0)	17 (11.0)	1.2 (0.6, 2.6)
Employee health policy	32 (20.5)	7 (4.5)	0.2 (0.1, 0.4)

### Sporadic *Salmonella* Cases

A total of 154 sporadic *Salmonella* case exposures in restaurants located in Bloomington and Richfield were identified from 2010 to 2015. This included 18 exposures in 2010 and 24 in 2015. One hundred and twelve exposures occurred from 2011 to 2014.

### Comparison of 2010 Survey to 2010 Sporadic *Salmonella* Case Inspections

In 2010, the FDA-based survey included 156 routine inspections, while 18 restaurants were identified that served a sporadic case of *Salmonella* (Table 2). Food contact surfaces were most frequently observed out-of-compliance in both the 2010 FDA-based survey (23.7%) and among sporadic *Salmonella* cases (50%). However, the rate of violations among the sporadic case restaurants was significantly higher (odds ratio [OR] = 3.2, 95% confidence interval [CI] = 1.2, 8.7). The only other risk factor that was more frequently out-of-compliance among sporadic case exposures was having handwash facilities stocked with hand cleanser, sanitary towels, or hand drying devices (OR = 2.8; 95% CI = 1.0, 7.8).

**Table 2:** Comparison of risk factor observations in 2010 FDA-based survey conducted by the City of Bloomington with inspections of restaurants that served a sporadic *Salmonella* case during 2010.

**FDA risk factor**	**2010 FDA-based survey (156 Food establishments)**	**2010 Sporadic *Salmonella* (18 Cases)**	**Comparison between *Salmonella* cases and survey**
	**No. (%) Inspections with observation out of compliance**	**No. (%) Inspections with observation out of compliance**	**Odds Ratio (95% confidence interval)**
Certified food manager	28 (17.9)	2 (11.1)	0.6 (0.1, 2.6)
Receiving conditions	1 (0.6)	1 (5.6)	9.1 (0.5, 152.5)
Cold holding	20 (12.8)	3 (16.7)	1.4 (0.4, 5.1)
Hot holding	10 (6.4)	1 (5.6)	0.9 (0.1, 7.1)
Time as a public health control	6 (3.9)	0	0 (0, 7.6)
Separate raw from ready to eat foods	18 (11.5)	0	0 (0, 1.9)
Food protected environmental contamination	5 (3.2)	0	0 (0, 9.9)
Food contact surfaces	37 (23.7)	9 (50.0)	3.2 (1.2, 8.7)
Proper, adequate handwashing	10 (6.4)	1 (5.6)	0.9 (0.1, 7.1)
Good hygienic practices	11 (7.1)	2 (11.1)	1.6 (0.3, 8.1)
Prevent contamination by hands	16 (10.3)	0	0 (0, 2.2)
Handwash facilities accessible	4 (2.6)	2 (11.1)	4.8 (0.8, 28.0)
Handwash facilities stocked	29 (18.6)	7 (38.9)	2.8 (1.0, 7.8)
Chemicals properly identified/stored/used	14 (9.0)	1 (5.6)	0.6 (0.1, 4.8)
Employee health policy	32 (20.5)	4 (22.2)	1.1 (0.3, 3.6)

### Comparison of 2015 Survey to 2015 Sporadic *Salmonella* Case Inspections

In 2015, the FDA-based survey included 155 routine inspections, while 24 restaurants were identified that served a sporadic case of *Salmonella* (Table 3). As in 2010, food contact surfaces were most frequently observed out-of-compliance in both the FDA-based survey (37.4%) and among sporadic *Salmonella* cases (58.3%) (OR = 2.4; 95% CI = 1.0, 5.7). Also, as in 2010, having handwash facilities stocked was more frequently out-of-compliance among sporadic case exposures (OR = 2.6; 95% CI = 1.1, 6.3). In addition, failing to have a food manager who has been certified in an accredited program, as determined by the Minnesota Department of Health, was observed for 10 (41.7%) sporadic case inspections compared to 18.1% of FDA- based survey inspections (OR = 3.3; 95% CI = 1.3, 8.1).

**Table 3:** Comparison of risk factor observations in 2015 FDA-based survey conducted by the City of Bloomington with inspections of restaurants that served a sporadic *Salmonella* case during 2015.

**FDA risk factor**	**2015 FDA-Based survey (155 Food establishments)**	**2015 Sporadic *Salmonella* (24 Cases)**	**Comparison between *Salmonella* cases and survey**
	**No. (%) Inspections with violation out of compliance**	**No. (%) Inspections with violation out of compliance**	**Odds ratio (95% Confidence interval)**
Certified food manager	28 (18.1)	10 (41.7)	3.3 (1.3, 8.1)
Receiving conditions	0 (0)	0	Undefined
Cold holding	45 (29)	4 (16.7)	0.5 (0.2, 1.5)
Hot holding	15 (9.7)	1 (4.2)	0.4 (0.1, 3.2)
Time as a public health control	14 (9.0)	1 (4.2)	0.4 (0.1, 3.5)
Separate raw from ready to eat foods	25 (16.1)	2 (8.3)	0.5 (0.1, 2.1)
Food protected environmental contamination	5 (3.2)	0	0 (0, 7.2)
Food contact surfaces	58 (37.4)	14 (58.3)	2.4 (1.0, 5.7)
Proper, adequate handwashing	8 (5.2)	1 (4.2)	0.8 (0.1, 6.7)
Good hygienic practices	5 (3.2)	0	0 (0, 7.2)
Prevent contamination by hands	10 (6.5)	1 (4.2)	0.6 (0.1, 5.7)
Handwash facilities accessible	24 (15.5)	6 (25.0)	1.8 (0.7, 5.1)
Handwash facilities stocked	38 (24.5)	11 (45.8)	2.6 (1.1, 6.3)
Chemicals properly identified/stored/used	17 (11.0)	2 (8.3)	0.7 (0.2, 3.4)
Employee health policy	7 (4.5)	11 (7.1)	0.9 (0.1, 7.8)

### FDA 2010 and 2015 Study and Sporadic *Salmonella* 2010 Through 2015 Case Study Comparison.

From 2010 through 2015, 154 food establishments were identified as food establishments that served sporadic *Salmonella* cases during the seven days before illness onset. The combined FDA-based studies in 2010 and 2015 included 311 routine restaurant inspections (Table 4).

**Table 4:** Comparison of risk factor observations in combined 2010 and 2015 FDA-based surveys conducted by the City of Bloomington with inspections of restaurants that served a sporadic *Salmonella* case from 2010 to 2015.

**FDA risk factor**	**Combined 2010 and 2015 FDA-based survey (311 Food establishments)**	**Combined 2010-2015 sporadic *Salmonella* (154 cases)**	**Comparison between *Salmonella* cases and combined surveys**
	**No. (%) Inspections with violation out of compliance**	**No. (%) Inspections with violation out of compliance**	**Odds ratio (95% Confidence interval)**
Certified food manager	56 (18.0)	23 (14.9)	0.8 (0.5, 1.4)
Receiving conditions	1 (0.3)	6 (3.9)	12.6 (1.5, 105.3)
Cold holding	65 (21.0)	39 (25.3)	1.3 (0.8, 2.0)
Hot holding	25 (8.0)	11 (7.1)	0.9 (0.4, 1.8)
Time as a public health control	20 (6.5)	5 (3.2)	0.5 (0.2, 1.3)
Separate raw from ready to eat Foods	43 (13.8)	5 (3.2)	0.2 (0.1, 0.5)
Food protected environmental contamination	10 (3.2)	2 (1.3)	0.4 (0.1, 1.8)
Food contact surfaces	95 (30.5)	78 (51.0)	2.3 (1.6, 3.5)
Proper, adequate handwashing	18 (5.8)	11 (7.1)	1.3 (0.6, 2.7)
Good hygienic practices	16 (5.1)	14 (9.1)	1.8 (0.9, 3.9)
Prevent contamination by hands	26 (8.4)	7 (4.5)	0.5 (0.2, 1.2)
Handwash facilities accessible	28 (9.0)	20 (13.0)	1.5 (0.8, 2.8)
Handwash facilities stocked	67 (21.5)	65 (42.2)	2.7 (1.7, 4.0)
Chemicals properly identified/stored/used	31 (10.0)	25 (16.2)	1.8 (1.0, 3.1)
Employee health policy	39 (12.6)	11 (7.1)	0.5 (0.3, 1.1)

As with the 2010 and 2015 comparisons, food contact surfaces (OR = 2.3; 95% CI = 1.6, 3.5) and handwash facilities stocked (OR = 2.7; 95% CI = 1.7, 4.0) were both found out-of- compliance more frequently among inspections of sporadic-case associated restaurants. In addition, receiving conditions of foods (OR = 12.6, 95% CI = 1.5, 105.3) and chemicals properly identified, stored, and used (OR = 1.8; 95% CI = 1.0, 3.1) were more likely to be cited at restaurants that served sporadic *Salmonella* cases. In contrast, separating raw from ready to eat foods (OR = 0.2; 95% CI = 0.1, 0.5) was less likely to be found out-of-compliance in restaurants that served sporadic cases of *Salmonella*.

## Discussion

The results from the FDA-based risk factor surveys showed increased numbers of inspections with out-of-compliance findings for cold holding, food contact surfaces and handwash facilities accessible from 2010 to 2015. In contrast, there was a marked decrease in violations for employee health policy, and non-significant reductions in out-of-compliance findings for several personal hygiene measures. This can be attributed to the interventions the City of Bloomington implemented after the 2010 risk factor analysis. The top three violations cited during each risk factor survey were briefed to the inspectors, and interventions were implemented and made a priority for inspectors to focus on during routine inspections, possibly resulting in inspectors not focusing on other violations in such detail. A focus was made on reducing employee health and personal hygiene risk factors to reduce food-handler associated transmission of foodborne illnesses. The increase of violations between 2010 and 2015, suggests the need for all risk factor violations to be a priority of inspectors and food establishments.

Although the FDA-based risk factor surveys provide useful feedback on trends in food safety practices and inspection priorities, the ultimate test of regulatory efforts is the reduction in the occurrence of foodborne illnesses. In this regard, the finding that violations such as food contact surfaces and handwash facilities being stocked, were more likely to be cited in sporadic.

*Salmonella* cases than during the FDA-based risk factor survey, suggests that these may contribute to an increased risk of *Salmonella* transmission in restaurants. Only 5–10% of *Salmonella* cases are associated with outbreaks. Thus, food handling errors that may not lead to sufficient contamination or amplification to produce an outbreak, could still result in sporadic exposures to *Salmonella* in these settings. The observed reductions in *Salmonella* in NY City following the introduction of letter grading, suggests this as well (6).

This study has several limitations. The FDA-based risk factor surveys represent a sampling of all inspections conducted at the beginning and end of the study period. These surveys were used because the inspection system used in the City of Bloomington, did not support aggregation and analysis of results of routine inspections. In the FDA-based survey, fast food and table service restaurants were separately sampled. In both the 2010 and 2015 surveys, table service restaurants were slightly overrepresented. Because table service restaurants tend to have more items found out-of-compliance on inspections, we believe that our combining of fast food and table service samples would have marginally biased our results toward the null. In addition, the practice of classifying observations as in compliance unless otherwise noted by inspection, leads to underestimation of the proportions of inspections with observations out of compliance. This may reduce observed measures of association, potentially biasing results toward the null. Finally, while we do not know the actual source of exposure for any of the sporadic *Salmonella* cases, results of numerous sporadic case control studies suggest that approximately half of sporadic cases likely had exposure outside of the home (8). Inclusion of sporadic cases due to non-restaurant exposures would have biased our results toward the null. Thus, the observed associations suggest that these may contribute to an increased risk of *Salmonella* transmission in restaurants. Given the multiple potential sources for introducing *Salmonella* into restaurant kitchens, and the ability of *Salmonella* to survive and grow in these settings, we believe that sporadic *Salmonella* cases make an important and unique indicator of the effectiveness of food safety practices in restaurants.

The results of this study warrant additional studies on the potential use of routine inspection reports as food safety hazard surveillance for restaurants, and the potential to link such hazard surveillance with other foodborne illness surveillance. Because restaurant inspections represent routine public health activities that are conducted by a broad range of agencies, establishing methods to analyze the results of these inspections across agencies and over time may help us better understand how risk factors cited on routine inspections can predict the likelihood a food establishment will be implicated in a foodborne illness case, in both sporadic cases and outbreaks. Unfortunately, documenting what food safety violations occur in restaurants doesn't explain why they occur. The Centers for Disease Control and Prevention's Environmental Health Services Branch has developed a National Environmental Assessment and Reporting System (NEARS) to document both what environmental factors contribute to outbreaks and why these factors occurred (9). This type of root-cause analysis is not available for routine inspections. However, the finding that food safety management characteristics are associated with fewer risk factor violations in routine inspections does suggest that active managerial control is important to develop and maintain food safety culture in restaurants (5).

## Author Contributions

XA, CH, and PL contributed conception and design of study, reviewed and revised manuscript. XA wrote the first draft of the manuscript. All authors contributed to the manuscript revision, read, and approved the submitted version.

### Conflict of Interest Statement

The authors declare that the research was conducted in the absence of any commercial or financial relationships that could be construed as a potential conflict of interest.
